# Self-Assembled Cysteamine Reporter Ligands for SERS Nitrate Detection in Continuous Flow

**DOI:** 10.1021/acs.langmuir.4c05378

**Published:** 2025-06-03

**Authors:** Timo Küster, Geoffrey D. Bothun

**Affiliations:** Department of Chemical, Biomolecular, and Materials Engineering, 4260 University of Rhode Island, 2 East Alumni Ave, Kingston, Rhode Island 02881, United States

## Abstract

Elevated nitrate concentrations in aquatic environments can contribute to the formation of harmful algal blooms, which lead to eutrophication. In this work, cysteamine self-assembled monolayers (SAMs) on two-dimensional gold nanostructured substrates were investigated for the capture and detection of nitrate anions by surface enhanced Raman scattering (SERS) under continuous fluid flow. An indirect detection strategy is demonstrated where cysteamine Raman activity and SAM reconfiguration change due to nitrate adsorption. Nitrate adsorption, as well as SAM reconfiguration based on the *gauche* to *trans* conformation ratio, were dependent upon the cysteamine protonation state. The terminating amine of cysteamine was Raman active when protonated near the expected SAM p*K*_a_ and the gold–thiol bond was increasingly Raman active above the expected p*K*_a_. Highly charged SAMs (pH 3) were not responsive to nitrate, suggesting that nitrate detection is reliant upon the dynamic interplay between protonation, charge state, and nitrate adsorption. Cysteamine SAMs responded to nitrate concentrations spanning 10^1^ to 10^3^ nanomolar (10^0^ to 10^2^ parts per billion), which are considerably lower than those previously reported for direct detection of nitrate using cationic SAMs. This work demonstrates the potential for indirect SERS detection of anionic pollutants using rationally selected capture + reporter ligands.

## Graphical abstract

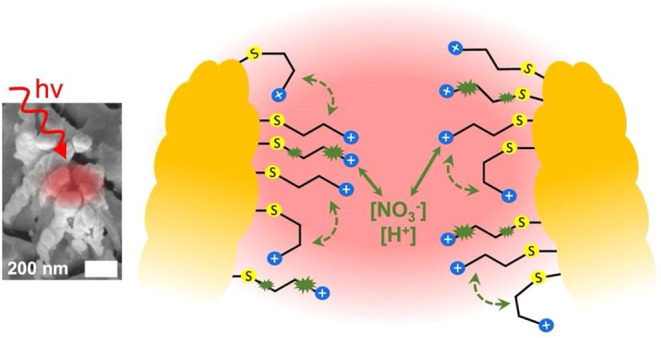

## Introduction

The detection of anionic environmental pollutants is a widely studied field due to the negative implications of elevated pollutant levels on human life and the environment.^1^ Elevated nitrate levels in aquatic environments, for example, can lead to harmful algae blooms and eutrophication. Significant attention has been given to using surface-enhanced Raman Spectroscopy (SERS) in ultrasensitive sensors to detect anionic molecules, including nitrate. SERS is a molecular fingerprinting technique relying on nanostructured plasmonic particles or surfaces such as gold (Au) or silver (Ag).^2,3^ SERS is theoretically capable of single molecular detection and can be utilized to monitor individual molecular bonds in near real time, making it an excellent tool to study the surface chemistry arising from analyte adsorption and substrate functionalization under varying conditions.^4–8^ In previous work, we demonstrated *in situ* detection of sodium nitrate by SERS based on symmetric and asymmetric nitrate vibrations attributed to image charge attraction to unfunctionalized gold nanopillar substrates.^9^ Spectral normalization against silicon, present in the SERS substrate, and laser orbital raster scanning were used to account for substrate variability, which can lead to significant differences in spectral data collected from replicate substrates.^3,10^

Cationic capture ligands, such as cysteamine below its p*K*_a_, have been used to increase the detection sensitivity for anionic pollutants. Cysteamine readily forms self-assembled monolayers (SAMs) on Au and Ag surfaces. In our recent work, we detected the anionic dye 5(6)-carboxyfluorescein through a charge transfer mechanism on cysteamine-functionalized SERS substrates with electrokinetic preconcentration.^11^ Direct SERS detection of the dye was achieved, but the Raman activity of cysteamine itself exhibited unique behavior, suggesting that indirect detection may also be achievable using cysteamine as both a capture and reporter ligand. Indirect detection would be based on the “activation” of cysteamine vibrational modes or changes in SAM stability under different aqueous conditions, including pH and the presence of anions.^12–16^ A prevalence of *trans* conformation, where the molecule is perpendicular to the surface forming a tightly packed SAM, to the *gauche* conformation, where the molecules lie down on the surface and disrupt SAM packing, is widely described in the literature and was also confirmed in our most recent publication.^11,13–15^ Cysteamine-functionalized SERS substrates have been used to directly detect nitrate (reporting a limit of detection of 10 μM or 620 ppb)^17^ as well as perchlorate anions,^18^ but in these studies, changes in the Raman activity of cysteamine as a reporter ligand were not considered.

In this study, we present a custom-designed millifluidic flow channel that was used to continuously measure the response of cysteamine-functionalized SERS substrates as a function of nitrate concentration and pH (Figure 1; additional details in Figure S1). Using a continuous flow system at laminar flow conditions ensured a constant nitrate concentration across the substrate, thereby (i) minimizing spatial variations in nitrate adsorption on the planar substrates and (ii) enabling SERS measurements in real time and without disturbances. Previous studies on the influence of different anions and solvents on alkanethiol SAMs focused on the formation of SAMs rather than monitoring the dynamic response of a SAM to changes in the surrounding medium after being formed.^8,14–16,18–26^ The latter is the goal of this work; to determine if the dynamic response of a cysteamine SAM can be used for the indirect detection of nitrate. Solution acidity (pH) was used to modify the charge state of cysteamine, and in this work, we show that pH values near the p*K*_a_ yield the most sensitive SAM.

**Figure 1:**
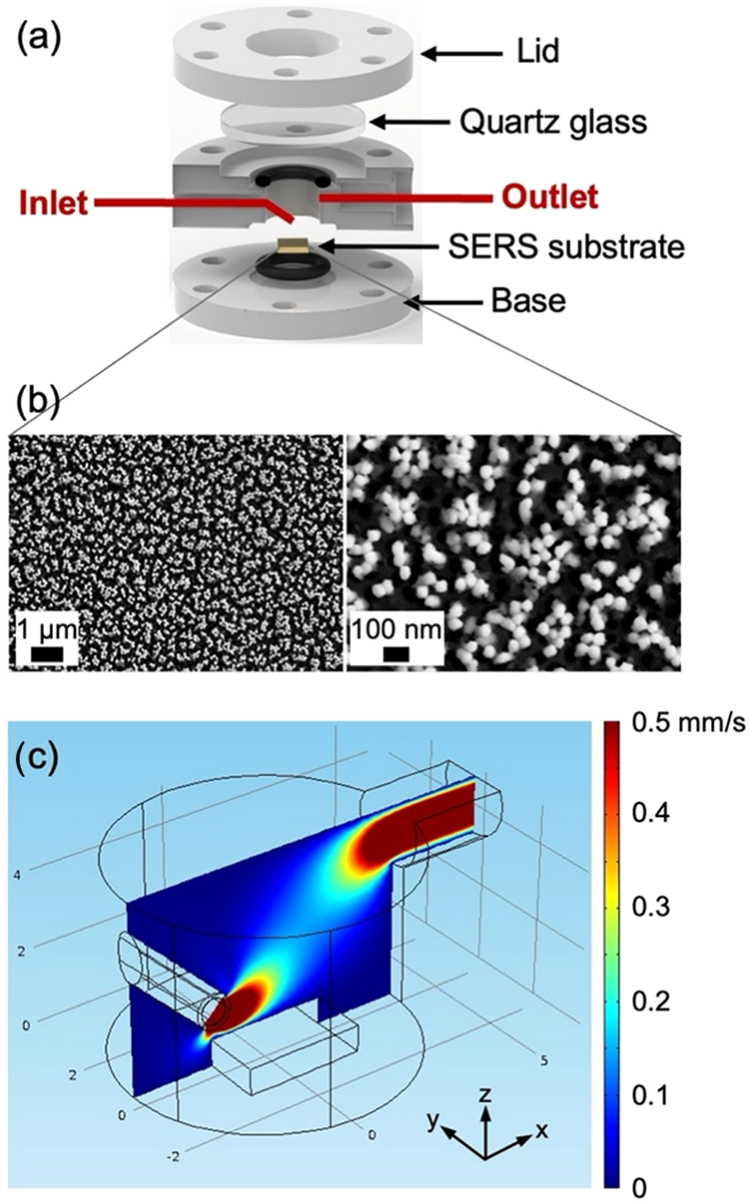
Continuous flow SERS device and substrate. (a) Render model showing the design and substrate location, (b) gold-capped silicon nanopillar SERS substrates after pillar collapse to form hotspots, and (c) COMSOL simulation of the flow chamber showing the flow path velocity field (shown in the heat map from 0 to 5 mm/s) through the device at an inlet flow rate of 80 μL/min.

## Experimental Section

### Materials

Cysteamine (95% purity) was purchased from Sigma-Aldrich and solutions for substrate functionalization were made with ACS/USP grade 200 proof anhydrous ethanol (Pharmco). Solutions were stored at 4 °C and used for 5 days. Nitrate solutions were made by dissolving sodium nitrate in deionized water (DIW) obtained from a Millipore system (18 MΩ) and stored at 4 °C. Solution pH was measured on a Thermo Scientific Orion Star A111 pH meter with a ThermoFisher microprobe and adjusted using NaOH or HCl solutions.

### Substrate Preparation and SAM Formation

Cysteamine SAMs were prepared on Gold SERSstrate (Silmeco ApS, Copenhagen, Denmark), as described previously.^11^ Briefly, SERS substrate preparation involved creating hotspots by leaning gold-capped silicon nanopillars together through capillary collapse, followed by oxygen-plasma cleaning to remove surface contamination. SAM was formed by immersing the substrates in 10 mM cysteamine for 24 h, rinsing with ethanol, and drying at room temperature for 30 min. The quality of the SAM was assessed by comparing Raman spectra of substrates before and after functionalization for the appearance and intensity of the characteristic cysteamine Raman peaks with focus on the *gauche* and *trans* CS modes at wavenumbers of 644 and 724 cm^–1^, respectively. SERS spectra were measured using a SIERRA 2.0 Raman spectrometer (Metrohm, WY) equipped with a 100 mW, 785 nm wavelength laser over an integration time of 5 s. Orbital raster scanning was used to yield an effective detection area of 2 mm in diameter. Baseline correction was carried out in accordance with previous reports.^9,11^

### SERS Measurements

SERS measurements were conducted under continuous flow using a custom device with a guided inlet stream, aimed toward the substrate surface to increase interaction of the substrate with the surrounding medium and improve mass transfer (Figure 1). Additional details of the SERS instrument and operation can be found in previous work.^9,11^ The inlet stream angle was optimized through COMSOL simulations at 60° from horizontal and a flow rate of 80 μL/min to provide close to constant analyte solution flow across the surface. The geometry of the assembled devices was confirmed using three-dimensional X-ray microscopy (3D-XRM; Figure S1). The channels were fabricated through additive manufacturing using a resin 3D printer.

## Results

### Cysteamine SAM Characteristics

Cysteamine in solution has three primary charge states: cationic, zwitterionic, and anionic, with increasing pH. When chemisorbed on a gold surface, two charge states are possible, cationic and neutral, where only the primary amine group can be protonated. The p*K*_a_ of a cysteamine SAM can vary greatly and depends on factors such as ionic strength and SAM density. A reasonable range of p*K*_a_ based on reported values is from pH 7 to 7.6,^26–28^ indicating that a majority of the cysteamine in a SAM would be positively charged below pH ∼7. In this case, it could be assumed that at a pH ≥ 10, a cysteamine SAM would be neutral. Three pH values were selected for this work to examine the SERS activity of a cysteamine SAM in a highly charged state (pH 3), in a moderately charged state near the p*K*_a_ (pH 6.8), and in a primarily neutral state (pH 10). While a highly charged SAM would provide the greatest potential to attract negatively charged analytes such as nitrate, it will also experience the greatest electrostatic repulsion between neighboring cysteamine molecules that will weaken the intermolecular van der Waals interactions that lead to high SAM density and stability

An in-depth characterization of the utilized substrates can be found in previous work.^9,11,29^ For these substrates, Raman signal enhancement is achieved through hotspots generated by preleaning gold-capped silicon nanopillars so the pillar tips are in close proximity (Figure 1b). Figure S2 shows a spectral comparison between functionalized and nonfunctionalized dry substrates, as well as a functionalized substrate that has been hydrated in excess water. Raman peaks at 644 and 724 cm^–1^ correlate with the *gauche* (*G*) and *trans* (*T*) configuration of the cysteamine C–S stretching mode, respectively, and demonstrate the successful functionalization of the SERS substrate with cysteamine.^11,13,14^ Additional peaks appear when cysteamine is in pure form (single granule; Figure S3) or as a SAM in a dry state (Figure S2), which are assigned in Table S1, and show good agreement with reported values.^13,14,30^

Plausible intermolecular interactions and SAM characteristics that will influence the SERS spectra are listed in Figure 2. The interactions and characteristics considered in this work include (i) nitrate binding to cysteamine via charge attraction or hydrogen bonding, (ii) cysteamine *trans* to *gauche* conformational changes, and (iii) intermolecular cysteamine interactions within a SAM (not depicted in Figure 2). These properties are interdependent and pH-dependent and expected to be dependent on nitrate concentration. The term “SAM activity” in this work describes the degree to which a cysteamine SAM responds to changes in pH and nitrate concentration. Changes in the intensity of specific cysteamine Raman bands caused by nitrate binding may occur in the *trans* or *gauche* ligand conformations. It is also expected that nitrate binding will influence transient changes in the SAM structure, reflected in the ratio of *gauche* to *trans* conformers. Lastly, in the *gauche* conformation, the Raman bands associated with the terminating primary amine group would become more intense as the amine group gets closer to the substrate surface and Au–N coordination bonds may form.

**Figure 2:**
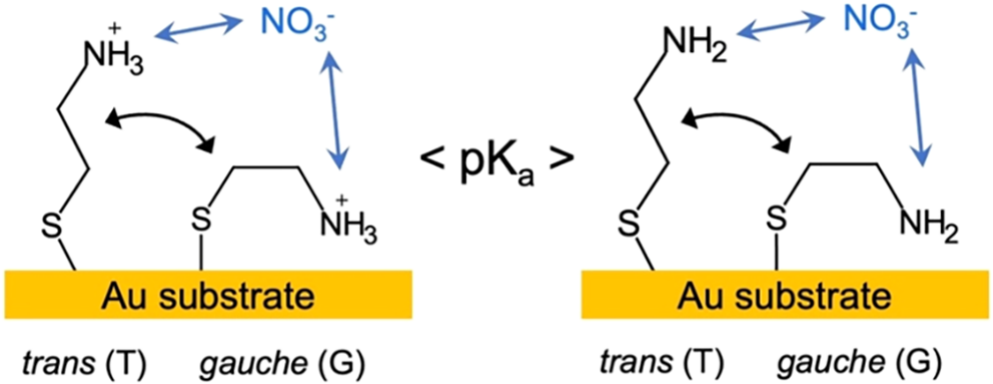
Schematic depicting plausible nitrate interaction with cysteamine and trans–gauche conformational changes of cysteamine ligands below (left) and above (right) the p*K*_a_.

### Influence of pH and Anions on Cysteamine SAM at High Nitrate Concentration

Measurements of cysteamine-functionalized SERS substrates were taken under continuous laminar flow conditions with and without the presence of dissolved sodium nitrate (100 μM) in deionized water (DIW). This high nitrate concentration, relative to the concentrations used to examine detection sensitivity, was chosen to quickly achieve substrate saturation with nitrate anions and focus on dynamic SAM behavior. Based on the flow rate of 80 μL/min and an internal volume of 165 μL, the residence time within the flow chamber was approximately 2.1 min, equating to 29 resident volumes passing through the chamber and over the SERS substrate each hour (Figure 1c). Representative spectra shown in Figure 3a,3b show distinct silicon peaks at 519 cm^–1^ originating from the Si contained within the substrates that was used to normalize the data.^9^ Changes in Si normalized Raman intensity were examined over several hours for cysteamine vibrational modes that span the length of the molecule and are associated with or adjacent to the sulfur atom or the thiol-gold bond. In solution, cysteamine exhibits (i) deformation of CSH at 1132 cm^–1^, (ii) deformation of CH_2_ @S at 1132 and 1451 cm^–1^, and wagging of the amine group, CH_2_ @N, at 1407 cm^–1^. It should be noted that Raman peaks for direct detection of dissolved nitrate were not observed at 1040 cm^–1^ as previously reported for a cysteamine SAM.^17^ In this work, a broad peak was observed for cysteamine from 1000 to 1050 cm^–1^ (Figure 1b) that masked the nitrate peak.

**Figure 3:**
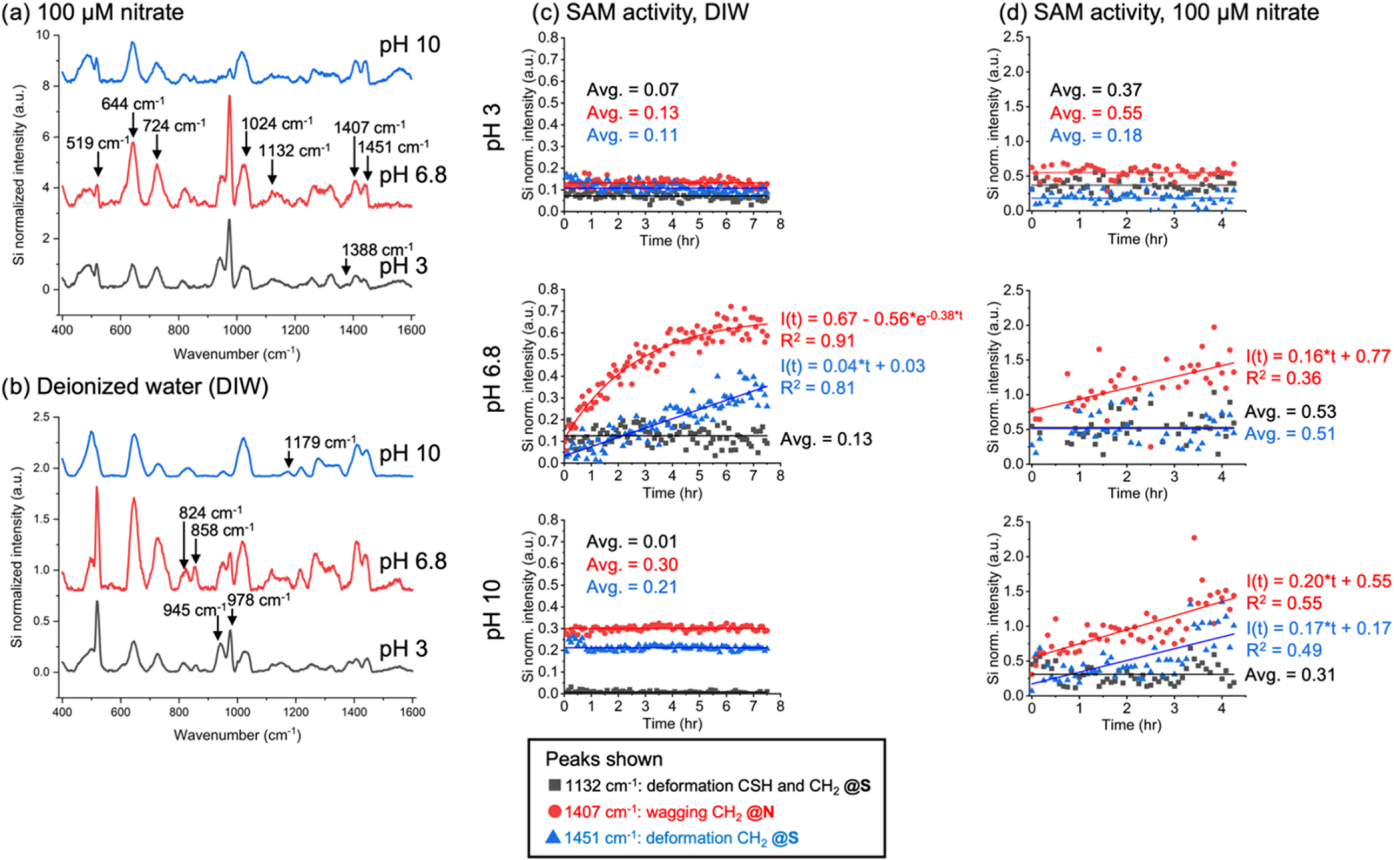
Representative SERS spectra (left panel) of a cysteamine SAM in (a) 100 μM sodium nitrate and in (b) deionized water (DIW) at pH 3, 6.8, and 10. The time dependency of the SERS intensity for selected peaks associated with cysteamine SAM^30^ are shown for (c) DIW (middle panel) and with (d) dissolved sodium nitrate (right panel); black: 1132 cm^–1^ deformation CSH and CH_2_ adjacent to S atom (CH_2_ @S), red: 1407 cm^–1^ wagging CH_2_ adjacent to N atom (CH_2_ @N), blue: 1451 cm^–1^ deformation CH_2_ adjacent to S atom (CH_2_ @S). All data shown are normalized by the intensity of the Silicon peak at 519 cm^–1^. The CSH assignment is based on comparison to cysteamine molecules in solution.

Results for SAM activity in DIW and in 100 μM nitrate are shown in Figure 2c,2d, respectively, at pH 3, 6.8, and 10. Some insightful observations arise from the dynamic changes in the intensity of the Raman peaks. First, in DIW (Figure 3c), the SAM is stable or “inactive” at pH 3 and 10 as depicted by the horizontal line fits representing time-averaged normalized intensity values. These pH values represent highly charged (≪p*K*_a_) and neutral (>p*K*_a_) states of the cysteamine ligands, respectively, suggesting that the pseudoequilibrium state of the SAM persists over extended periods of time under these conditions. Dynamic changes are observed at pH 6.8 for the 1407 cm^–1^ (CH_2_ @N; logarithmic growth) and 1451 cm^–1^ (CH_2_ @S; linear growth) peaks that correspond to increased SAM activity at a pH slightly below the expected p*K*_a_. Second, the normalized Raman intensity in the presence of 100 μM nitrate (Figure 3d) is higher for all vibrational modes at equivalent pH values and time points. The data points are also more scattered compared to DIW leading to greater uncertainty in the linear (horizontal) averages and in the linear growth line fits for the CH_2_ @N peak at 1407 cm^–1^ (pH 6.8 and 10) and the CH_2_ @S peak at 1451 cm^–1^ (pH 10). Third, at pH 3 with 100 μM nitrate present, where the highest binding would be expected due to charge attraction (NH_3_^+^–NO_3_^–^), the three normalized Raman peak intensities examined did not change with time, indicating surface saturation. Fourth, at pH 10 the SAM is inactive in DIW but active in 100 μM nitrate based on the CH_2_ @N and CH_2_ @S peaks. Salts are known to reduce the p*K*_b_, or increase the p*K*_a_, of strong bases, and the SAM activity observed at pH 10 may be due to partial protonation of cysteamine leading to nitrate adsorption. Alternatively, nitrate binding at pH 10 may resulted from hydrogen bonding to neutral cysteamine. These observations suggest that the dynamic SAM response occurs at pH values near p*K*_a_ in the absence or presence of nitrate anions.

Additional insight can be gained by analyzing other Raman bands in the spectra (Figure 3a,3b). A “double peak” is present between 800 and 860 cm^–1^ for all spectra except water at pH 10. Raman bands at the lower end of this range result from rocking motions or torsion of methylene groups at the amine and thiol groups.^30,31^ Riauba et al.^30^ report peaks at 817 and 824 cm^–1^ associated with methylene vibrations for dissolved cysteamine as a zwitterion or anion, respectively, however, these ionized forms would not be present in a thiol-bonded SAM. The peak at 858 cm^–1^ is likely due to the deformation of CCN groups^30^ or wagging of amine groups^31^ within the SAM. Depending on the conditions these double peaks present differently: for water at pH 3 the peak near 824 cm^–1^ is higher than the 858 cm^–1^ peak and for pH 6.8 the opposite is observed, meaning that a decrease in pH causes an increase of the rocking motion or torsion of the SAM with respect to the deformation. In the presence of nitrate, the peak intensity near 824 cm^–1^ decreases with decreasing pH with respect to the 858 cm^–1^ peak intensity, and therefore, with decreasing pH the rocking motion subsides. This could be due to the presence and interaction of nitrate with the SAM. As a positive SAM charge and therefore attraction of nitrate anions is expected at lower pH, a decrease in rocking motion can be explained through a stabilization of the SAM through nitrate charge saturation. In the absence of nitrate, this stabilization mechanism is not possible, which explains the comparatively higher activity of the peak near 824 cm^–1^ for pH 3 in the absence of nitrate.

The region from 940 to 1070 cm^–1^ shows increased intensity. For all conditions except nitrate at pH 10, a CNH deformation and rocking peak are present at 945 cm^–1^. A sharp peak at 978 cm^–1^ is present for pH 3 and 6.8 with and without the presence of nitrate. The same peak is absent for water at pH 10 and is very small in comparison to the lower pH values in the presence of nitrate. This peak was observed by others,^13^ but the origin of this peak is unclear. A broad peak associated with asymmetric stretching of CCN is present for all conditions at a wavenumber of 1024 cm^–1^. The investigation of these peaks shows the inherent Raman activity of the CCN “spine” of the molecules, making up the SAM, and the consistent intensity of the 1024 cm^–1^ peak shows the continuing presence of a cysteamine SAM, despite the formation of *gauche* defects as described below.

A wide, convoluted peak is observed for all spectra, except for water at pH 10, between wavenumbers 1120 to 1179 cm^–1^. For water at pH 10, a single peak at 1179 cm^–1^ is present. The assignment of this region indicates a twisting motion of the amine headgroup of the SAM, as well as deformation of the CS group and twisting of the associated methylene group. The narrower band present for water at pH 10 could be due to a more limited range of motion of the SAM, which is most likely caused by the protonation of the SAM under these conditions.^30^ Twisting of the amine headgroup is further revealed in water at pH 3, as well as nitrate solutions at pH 3 and pH 6.8, based on a small shoulder present at 1388 cm^–1^, suggesting that under these conditions, the amine groups of the SAM that are exposed to aqueous solutions show higher degrees of freedom for this motion, which again is likely due to the presence of nitrate anions.

Analysis of the vibrational modes of cysteamine clearly shows that pH and nitrate anions, and the combination of the two, affect the dynamic activity of the SAM. The presence of nitrate increases the activity of Raman modes associated with the SAM headgroup, showing that interactions occur even though no direct nitrate signal is observed at the concentrations investigated here. Interactions with or within a cysteamine SAM can be assessed by monitoring the development of *gauche* “defects” based on the ratio (*G*/*T*) of the characteristic peak intensities of C–S stretching at 644 cm^–1^ (G) and 724 cm^–1^ (T).^14,32^ For example, at pH 6.8 in deionized water, G/T was initially 0.75 and increased to 2 after 440 min (Figure 4a). The G/T range is consistent with previous work by Marmisollé et al.^32^ where 1.78 was reported for a cysteamine SAM on silver electrodes, and by Michota et al.^14^ where ∼1 was measured on gold electrodes.

**Figure 4:**
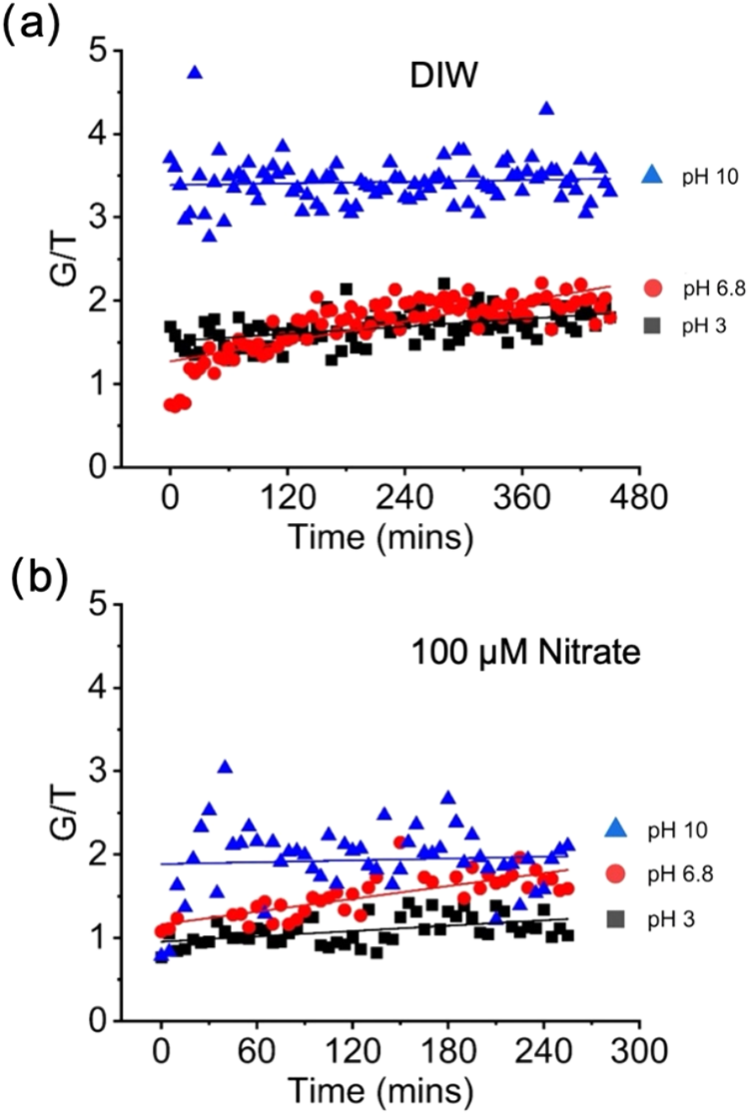
Changes in the cysteamine SAM *gauche* (G) to *trans* (T) peak intensity ratio, G/T, as a function of pH in (a) deionized water (DIW) and (b) in the presence of 100 μM sodium nitrate.

All parameters tested led to dynamic changes in the G/T ratio with an increasing *gauche* conformation. Comparing the time dependency of the G/T intensity ratio for pH 3 with (Figure 4b) and without (Figure 4a) nitrate present, we see the *gauche* conformation prevalence increasing linearly with time at identical rates (slope of best fit line = 0.07). The presence of 100 μM nitrate shifts the linear best fit downward by a ratio difference of 0.55 (Y-intercept of 1.45 vs 0.90, respectively), meaning that in this case the presence of nitrate at low pH has reduces the *gauche* confirmation or stabilizes the SAM by promoting the *trans* conformation that enables greater ligand packing. At a pH of 6.8 with and without the presence of nitrate, the trend is similar and shows increases in the G/T ratio with time with little difference in the G/T ratio. With a lower prevalence of protonated cysteamine at pH 6.8 compared to pH 3, the influence of nitrate is expected to be lower at pH 6.8 due to less nitrate adsorption. A significant difference in G/T is observed at pH 10. In DIW, the G/T ratio remains near 3.5, while with 100 μM nitrate, the ratio remains near 2 (excluding data near time zero). These results at a high nitrate concentration suggest stabilization of the SAM, inferred by fewer *gauche* defects, caused by nitrate adsorption that neutralizes the charge on cysteamine and/or reduces coordination bonding between the cysteamine nitrogen and the gold surface (Au–N). The G/T ratios at pH 10 further support the assertion that nitrate adsorbed to and increased the p*K*_a_ of the cysteamine SAM.

### Influence of pH and Anions on Cysteamine SAM at Low Nitrate Concentrations

The effects of lower nitrate concentrations are now examined (i) to elucidate the interaction mechanism between the nitrate anion and the SAM and (ii) to correlate SAM Raman activity with nitrate concentration. While for the high nitrate concentration the dynamic behavior was examined, here the data for each nitrate concentration (10 nM to 1 μM for pH 3; 50 nM to 2 μM for pH 6.8 and 10) is collected continuously over 60 min and reported as averages with standard deviation. The influence of nitrate concentration and solution pH on the Raman activity of cysteamine SAMs was examined by comparing full spectra (Figure 5a–c) and selected peaks (Figure 5d–f; same peak selection shown in Figure 5c,d) that were identified to correlate with the cysteamine amine headgroup as a function of anion concentration. At pH 3, corresponding to the greatest positive surface charge of the SAM, the normalized Raman intensity changed in response to low nitrate concentrations with the intensity of the wagging mode of the CH_2_ group closest to the amine headgroup (CH_2_ @N) increasing slightly and the intensity of the deformations of the CH_2_ group of the thiol-gold bond decrease with increasing nitrate concentration. However, these changes were modest compared to the standard deviation and do not reflect a responsive or ‘active’ SAM, as observed at high nitrate concentration (Figure 3d).

**Figure 5:**
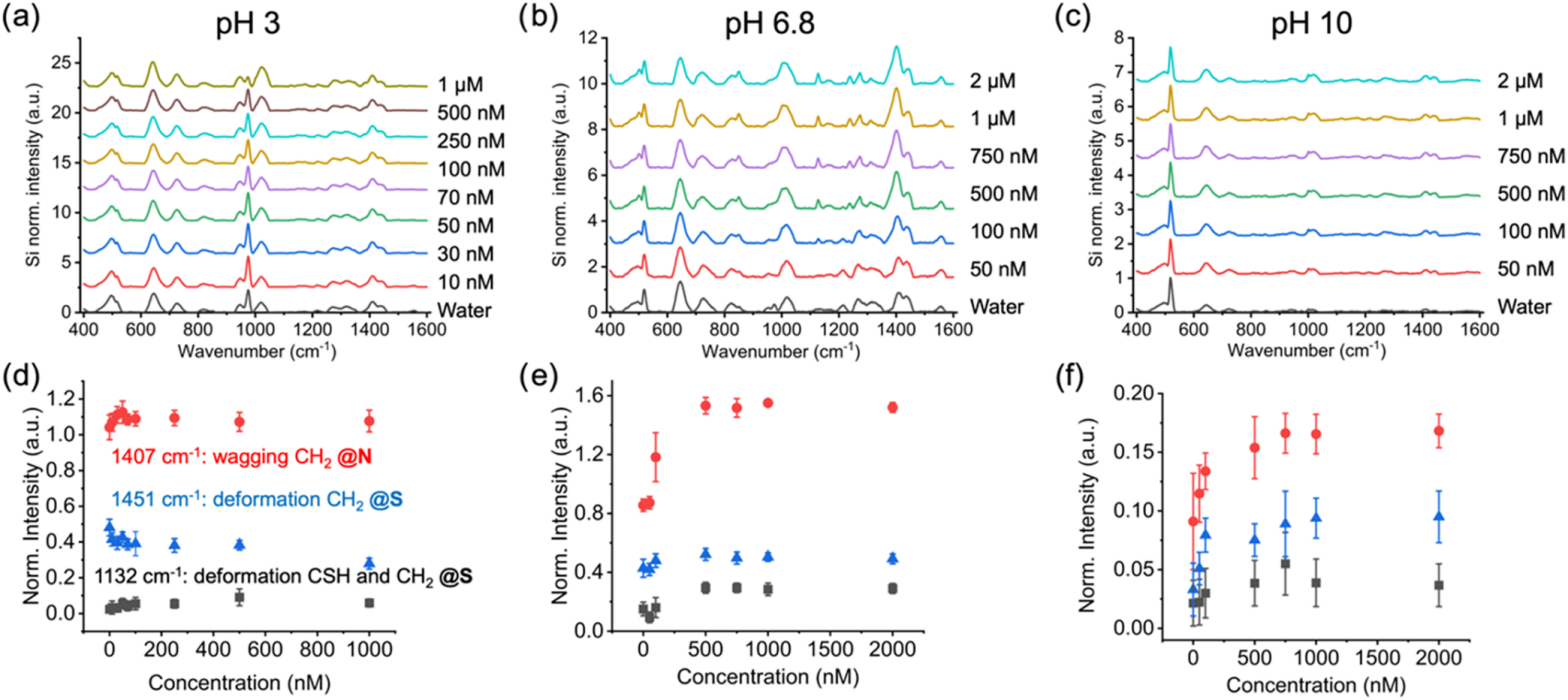
Spectra of cysteamine SAM under varying pH values and nitrate concentrations (a) pH 3; concentration 0–1 μM (b) pH 6.8; concentration 0–2 μM (c) pH 10; concentration 0–2 μM (d) pH 3; influence of pH and nitrate concentration on selected peaks,^30^ (d) pH 3; concentration 0–1 μM, (e) pH 6.8; concentration 0–2 μM, and (f) pH 10; concentration 0–2 μM.

SAM activity was the greatest at pH 6.8 in response to nitrate concentration and this activity corresponded to the CH_2_ @N peak at 1407 cm^–1^ nearest to the site of nitrate adsorption – the protonated primary amine headgroup (Figure 4e). The remaining Raman modes at CS and CH_2_ @S were less sensitive to the nitrate ion. Additional characteristic Raman modes became apparent at pH 6.8 that were not previously discussed or apparent at the high nitrate concentration. For example, at nitrate concentrations >50 nM, a peak at 1211 cm^–1^ disappears and a new peak appears at 1237 cm^–1^, both of which are associated with CH_2_ twisting close to the sulfur–gold bond. The convoluted peak from 1240 to 1360 cm^–1^, with a maximum at 1268 cm^–1^ (wagging CH_2_ @S and twisting of the amine group) deconvolutes to two peaks at a nitrate concentration ≥500 nM (1268 cm^–1^ shifts to 1272 cm^–1^ and 1309 cm^–1^ becomes distinct).^30^ Most of these previously unseen peaks are correlated with Raman modes close to the thiol-gold bond, suggesting that the cysteamine SAM gains additional degrees of freedom for vibrational modes close to the sulfur–gold bond due to SAM destabilization with increasing nitrate anion concentration.

At pH 10, the overall Raman intensity is significantly lower compared to the more acidic conditions (Figure 5f). This low intensity gave rise to greater experimental uncertainty in the 60 min time-averaged G and T peak intensities. However, the SAM is active with increasing nitrate concentration, with CH_2_ @N showing the greatest response followed closely by CH_2_ @S. Like the results at pH 6.8, the CH_2_ @N deformation intensity begins to plateau at a nitrate concentration of 500 nM, again supporting the concept that the SAM p*K*_a_ shifted to higher pH.

Lastly, we consider the presence of *gauche* defects based on the G/T ratio at low nitrate concentrations. At pH 3, an initial decrease in G/T ratio can be observed, which means that the SAM is reorienting itself with cysteamine adopting a more *trans* conformation below 100 nM nitrate (Figure 6a). Above this nitrate concentration, the cysteamine becomes neutralized due to nitrate adsorption based on the increase of the rocking peak (824 cm^–1^) for the CH_2_ groups adjacent to the N and S atoms. This determination is based on the work of Riauba et al.,^30^ where the 824 cm^–1^ peak was correlated with a zwitterionic charge state of cysteamine in solution, but not with a positively charged state. At pH 6.8 and 10, the expected increase of the G/T ratio with increasing nitrate concentration is observed (Figure 6b,c). The large uncertainty in the time-averaged intensities at these pH values reflects a dynamic G to T restructuring due to the proximity of pH to the assumed or shifted p*K*_a_ and to nitrate adsorption at cationic binding sites.

**Figure 6:**
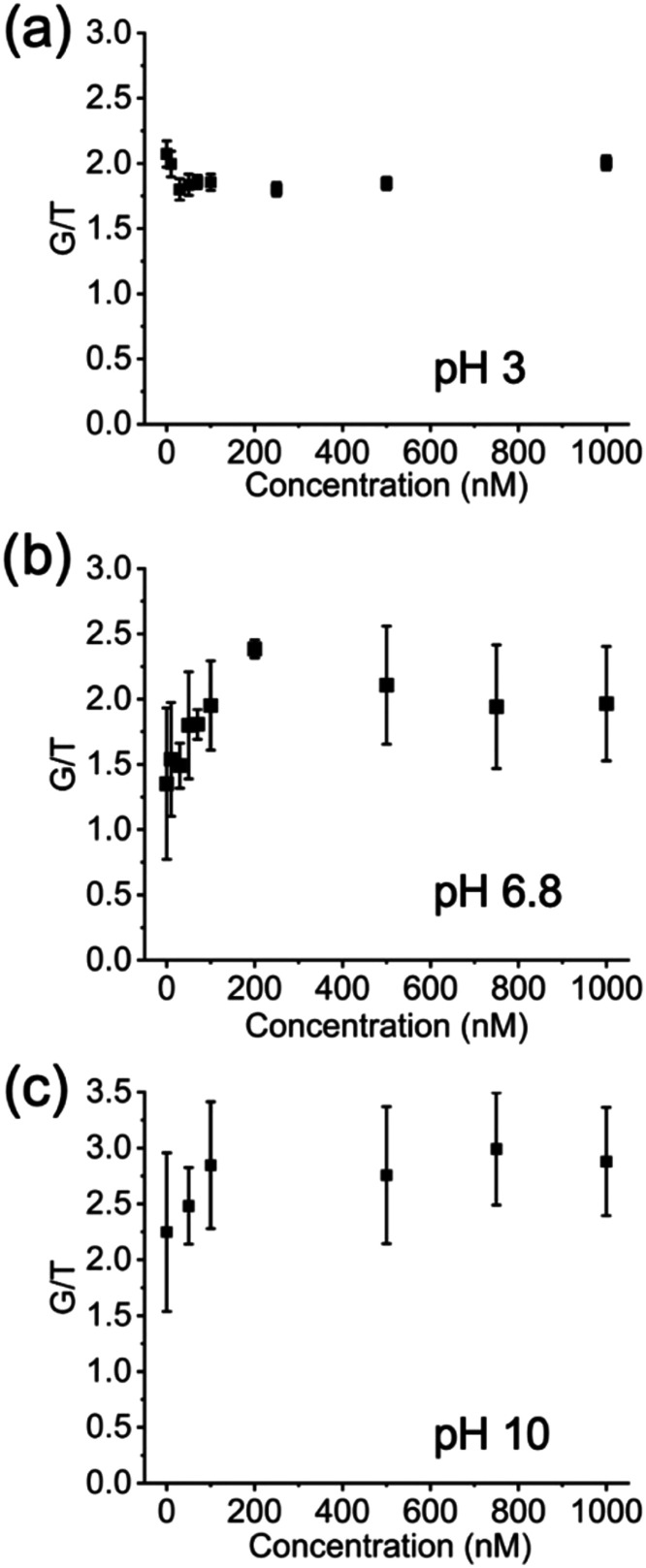
Changes in the cysteamine SAM *gauche* to *trans* peak intensity ratio, G/T, as a function of pH and nitrate concentration from 10 nM to 1 μM. Standard deviation represents time-averaged peak intensity measurements over 60 min for a given nitrate concentration.

## Conclusions

A detailed analysis of the effects of pH and nitrate anions on cysteamine vibrational modes and SAM conformation shows that cysteamine is best suited as a detection reporter ligand at pH values near p*K*_a_ where there is a dynamic interplay between protonation and nitrate adsorption. Collectively, the results support the proposed interactions depicted in Figure 2. The vibrational mode nearest to the cationic amine binding site was most sensitive to nitrate concentrations from 50 to 500 nM (3.1 to 31 ppb), and structural SAM changes based on *gauche* and *trans* conformations were sensitive to nitrate concentrations ranging from 10 to 200 nM (0.6 to 12.4 ppb). The levels of *indirect* nitrate detection are much lower than reported values for *direct* nitrate detection using a cysteamine SAM where a 10 μM (620 ppb) limit of detection was reported.^17^ We speculate that the enhanced sensitivity of the *indirect* approach may stem from the charge density distribution among cysteamine, the gold surface, and the nitrate solution. By introducing increasing concentrations of nitrate anions, the electron density distribution shifts toward the amine headgroup/liquid interface and leads to SAM polarization that activates Raman modes.

## Supplementary Material



## Notes

Continuous flow SERS device; Raman spectra of SERS substrate and cysteamine granulate; and cysteamine Raman mode assignments (PDF)
