# Supplementary material for: Self-Assembled Cysteamine Reporter Ligands for SERS Nitrate Detection in Continuous Flow
Source: Langmuir. 2025 Jun 3;41(23):14669–76. doi: 10.1021/acs.langmuir.4c05378 (PMC12422535; doi:10.1021/acs.langmuir.4c05378)
Supplement: Supplementary file 1 [file la4c05378_si_001.pdf]

Supporting information

Self-assembled cysteamine reporter ligands for SERS nitrate detection in continuous flow

Timo Küster and Geoffrey D. Bothun*

Department of Chemical Engineering, University of Rhode Island, 2 East Alumni Ave, Kingston, RI, 02881 USA

*Corresponding author: gbothun@uri.edu, +1-401-874-9518

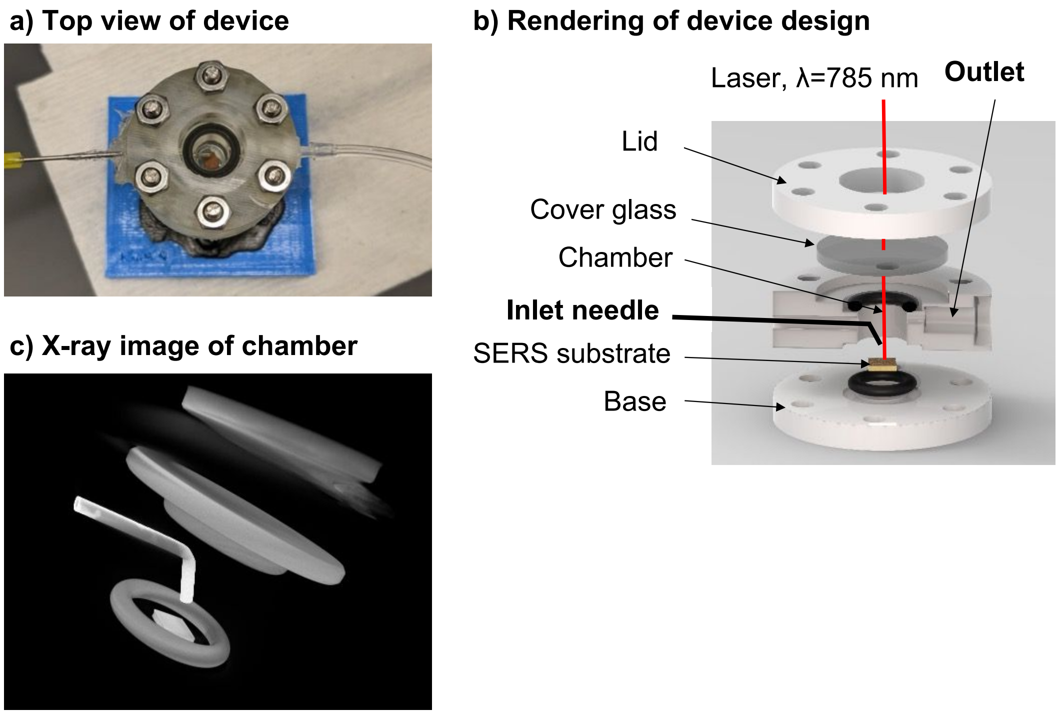

Figure S1. Continuous flow SERS device. a) Top view of device showing inlet (left) and outlet (right) flow paths and the SERS substrate (center). b) Computer rendering of the device. c) X-ray microscope (Zeiss Xradia Versa 610 XRM) image showing the internal 3D structure of the device partially disassembled.

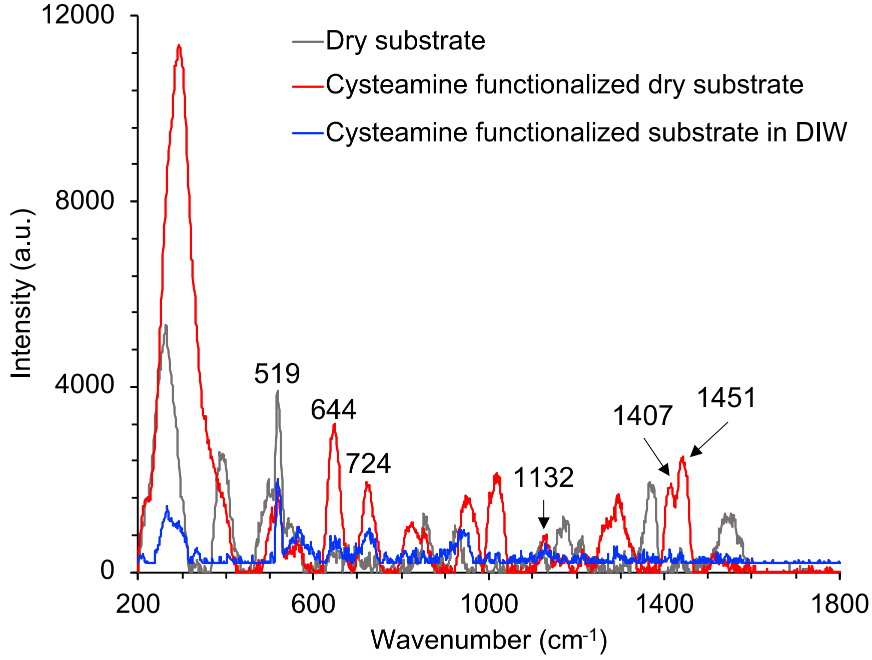

Figure S2. Raman spectra of SERS substrate before (gray) and after functionalization (dry = red; in DIW = blue).

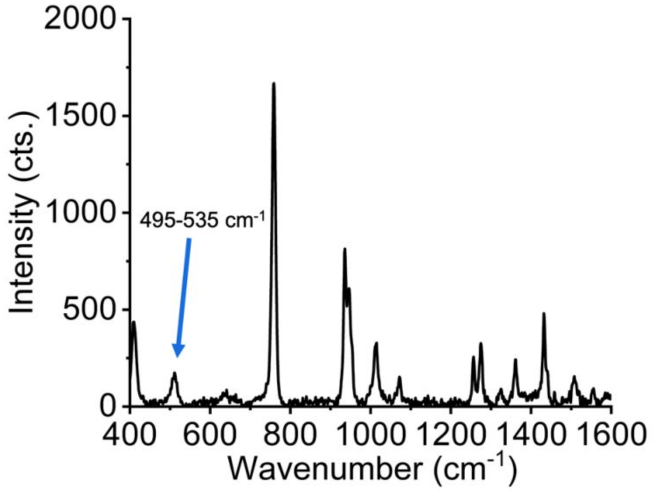

Figure S3. As received cysteamine granulate. Normal Raman measurement, 10 s integration time.

Table S1. Cysteamine Raman mode assignments based on Riauba et al.[1]

**Wavenumber (cm^{-1})**	**Vibrational assignment**
641, 663	CS stretching
644	CS stretching (gauche)
724	CS stretching (trans)
824	CH_2 (adjacent to S and N), rocking
858	CSH and CNH, deformation
943	CNH deformation, NH_3 rocking
978	unassigned
1024	CCN asymmetric stretching
1132	CSH and CH_2 (adjacent to S), deformation
1234	CH_2 (adjacent to S), twisting
1268	CH_2 (adjacent to S), wagging; NH_2, twisting
1281	CH_2 (adjacent to S), wagging
1331	CH_2 (adjacent to N), twisting
1384	CH_2 (adjacent to N) and NH_2, twisting
1388	CH_2 (adjacent to N), wagging
1407	CH_2 (adjacent to N), wagging
1451	CH_2 (adjacent to S), deformation
1464	CH_2 (adjacent to N), deformation<br>NH_3 symmetric deformation

References

1. Riauba, L.; Niaura, G.; Eicher-Lorka, O.; Butkus, E., A Study of Cysteamine Ionization in Solution by Raman Spectroscopy and Theoretical Modeling. *J Phys Chem A* **2006**, *110*, 13394-404.
